# A new anisotropy index on trabecular bone radiographic images using the fast Fourier transform

**DOI:** 10.1186/1471-2342-5-4

**Published:** 2005-05-31

**Authors:** Barbara Brunet-Imbault, Gerald Lemineur, Christine Chappard, Rachid Harba, Claude-Laurent Benhamou

**Affiliations:** 1 Equipe Inserm 658, Hôpital Porte Madeleine, BP 2439, 45032 Orléans Cedex 1, France; 2 Laboratoire d'Electronique, Signaux, Images, Université d'Orléans, BP 6744, 45067 Orléans, France

## Abstract

**Background:**

The degree of anisotropy (DA) on radiographs is related to bone structure, we present a new index to assess DA.

**Methods:**

In a region of interest from calcaneus radiographs, we applied a Fast Fourier Transform (FFT). All the FFT spectra involve the horizontal and vertical components corresponding respectively to longitudinal and transversal trabeculae. By visual inspection, we measured the spreading angles: Dispersion Longitudinal Index (DLI) and Dispersion Transverse Index (DTI) and calculated DA = 180/(DLI+DTI). To test the reliability of DA assessment, we synthesized images simulating radiological projections of periodic structures with elements more or less disoriented.

**Results:**

Firstly, we tested synthetic images which comprised a large variety of structures from highly anisotropic structure to the almost isotropic, DA was ranging from 1.3 to 3.8 respectively. The analysis of the FFT spectra was performed by two observers, the Coefficients of Variation were 1.5% and 3.1 % for intra-and inter-observer reproducibility, respectively. In 22 post-menopausal women with osteoporotic fracture cases and 44 age-matched controls, DA values were respectively 1.87 ± 0.15 versus 1.72 ± 0.18 (p = 0.001). From the ROC analysis, the Area Under Curve (AUC) were respectively 0.65, 0.62, 0.64, 0.77 for lumbar spine, femoral neck, total femoral BMD and DA.

**Conclusion:**

The highest DA values in fracture cases suggest that the structure is more anisotropic in osteoporosis due to preferential deletion of trabeculae in some directions.

## Background

The cancellous bone microarchitecture corresponds to the spatial organization and morphology of the trabecular network. Sugita *et al*. [1] observed that the mechanical behavior of the cancellous bone varied following the testing direction; these variations were interpreted as an anisotropic feature of the bone stress. Trabecular bone anisotropy corresponds to the preferential orientation(s) of trabeculae. Anisotropy is constituted under the influence of prefererential oriented strength applied to bone [2] and permits to establish resistance to these strengths in a given preferential direction. Sugita *et al*. [1] concluded that anisotropy of the cancellous bone should be considered to predict the fracture risk.

The determination of collagen and crystal orientation in connective tissues at molecular scale has been studied, for instance by diffraction [3-6]. Amorphous materials such as reinforced concrete have been characterized by x-rays and the orientation of fibers investigated by the Fourier transform method [7]. Defossez et al. assessed several methods on images of the femoral neck region: co-occurrence and run length matrix, spatial-frequency and fractal techniques to determine the trabecular direction [8]. These methods exhibited a high degree of accuracy, suggesting a possibility of trabecular orientation characterization in clinical practice [8].

Different methods are available to characterize the structural anisotropy on bone radiographs. In 1970, Singh *et al*. [9] developed a semi-quantitative index applied to femoral neck radiographs. This index is based on the existence of several arches of trabeculae in the femoral neck. Some arches preferentially disappear with age and osteoporosis, and the count of these arch systems can be used to determine the Singh index. Aggarwal *et al*. [10] developed a similar index for the calcaneus. Whitehouse *et al*. [11] introduced the Mean Intercept Length (MIL) defined as the average length between two bone/marrow interfaces. The MIL polar diagram generates an ellipse for 2D slice images [11] or projectional images such as radiographs [12], and an ellipsoid for 3D images [13]. A MIL tensor can be calculated [13] by fitting the MIL values and can be used to determine the Degree of Anisotropy (DA) [14]. The MIL method requires a previous binarization of the gray-level images, then it is a suitable method for the calculation of DA from 3D images obtained by Quantitative Computed Tomography (QCT) [15,16] or Magnetic Resonance Imaging [17]. Therefore, the binarization on 2D radiographs remains a critical step due to partial volume effect. The MIL method was used by Luo *et al*. [18] to compare the anisotropy of the 3D structure and of the 2D projected image of one *in vitro *sample and 14 simulation models derived from this sample. The authors reported a very good correlation (r = 0.99) between anisotropy values assessed on the 3D trabecular structure and the 2D projection images. Luo *et al*. [18] insisted on the interest of the estimation of architectural fabric from plain radiographs.

Few methods evaluating the trabecular structure have been developed on radiographs. Caldwell *et al*. [19] developed a method characterizing trabecular bone structure on vertebral digitized radiographs. An evaluation of the orientation named the fraction orientation edges is obtained from the histogram of the magnitude of the edge gradient versus the direction of the edges. Geraets *et al*. [20] developed an index called Line Fraction Deviation (LFD) derived from the tresholded radiographs and based on the fraction of bone pixels. A diagram plotting the standard deviation of this fraction in all directions could reflect the orientation of the trabecular bone. This method has been found more sensitive than MIL method on bone radiographs to evaluate anisotropy [12]. Jiang *et al*. [21] developed a method for a non invasive evaluation of bone mechanical properties: textural features, the global Minkowski dimension and trabecular orientation were determined using Minkowski dimension analysis. Jiang *et al*. demonstrated the contribution of normalized Bone Mineral Density (BMD), structural features and patient age to bone mechanical properties. In 1993, Oxnard reported slight visible microarchitectural changes from the Fourier transform on bone radiographic images but no parameter calculation was performed [22]. The same group worked on vertebral bodies T1 to L5 in seven male columns and applied the FFT to the radiographic images of these vertebrae. They characterized the orientation and size of the architectural elements of vertebral cancellous bone, and underline the potential of this technique to define the effects of ageing [23]. Wigderowitz *et al*. considered the properties of Fast Fourier Transform (FFT) to evaluate trabecular bone structure: the directional pattern of frequencies with high-magnitude can identify the orientation of trabeculae. He defined three indices including spectral trabecular index, longitudinal and transversal trabecular indices [24]. These indices approximate the evaluation of the trabecular structure in terms of trabeculae spacing and orientation. Wigderowitz *et al*. concluded that this quantification detects structural changes occurring with age and may be useful in osteoporosis studies [24]. Caligiuri *et al*. [25] also used the FFT to perform a structural analysis of the spine trabecular bone. The hypothesis was that the magnitude and the first moment of the power spectrum could correspond to the coarseness or the fineness of the texture pattern. The two texture measurements were compared to BMD. Caligiuri *et al*. concluded that this texture analysis might lead to a better prediction of the osteoporotic fracture risk [25]. Recently, Gregory et al. have proposed a FFT-based analysis of trabecular bone structure on hip radiographs from patients with and without hip fracture. They worked on FFT profiles parallel and perpendicular to the preferred orientation. Principal conponents analysis was used to generate scores from the profiles and was able to discriminate fracture and control groups better than fractal dimension [26].

Our research group has previously experienced fractal analysis on bone radiographs [27-29]. The measured parameter (Hurst coefficient) offers a global evaluation of the image irregularity, of its complexity. It is considered as an indicator of roughness. The Maximum Likelihood Estimator is applied first on one direction, then the process is repeated in 36 directions by 10 degrees steps. The directional results can be expressed on a polar diagram that can be itself be fitted to an ellipse; the shape of this polar diagram can be characterized in order to determine the textural anisotropy [30,31]. When comparing radius and calcaneus in the same subjects, Lespessailles *et al*. have shown significant differences [31] but the interpretation is equivocal because this technique characterizes the variations of grey level roughness following directions, and not directly the trabecular network orientation.

The osteoporotic fracture risk prediction is important especially in postmenopausal women. BMD is currently measured in clinical practice and textural parameters can be assessed on trabecular bone radiographs [19-22,24,25,31], but the anisotropy was not clearly quantified in these texture analyses. To address this question, we have worked on a quantitative evaluation of anisotropy on bone radiographs. The development of a structural parameter such as DA from radiographs is very promising; this parameter could be complementary of the BMD and of fractal texture parameters in the explanation of bone strength.

We describe here a new quantitative method based on the FFT to obtain anisotropy indices on bone radiographs of the calcaneus and a validation on synthetic images. The intra and inter-observer reproducibility and a pilot clinical study comparing osteoporotic fracture cases to control cases are presented.

## Methods

### Population of the pilot study

Postmenopausal women were recruited from a cross-sectional unicenter case control study. The protocol screened 400 women: 349 were enrolled. The inclusion and exclusion criteria have been previously described in details [28]. For this preliminary study, a sample of 22 osteoporotic fracture cases was randomly selected, and was secondarily age-matched with 44 control cases (for each age, we randomly selected two control cases with an equal age ± 5 years). The distribution of fracture cases was 1 hip fracture, 5 wrist fractures, 11 vertebral fractures and 5 metatarsus fractures, all these fractures were considered as low energy fractures. The ages of the control and fracture cases ranged from 45 to 87 years. The mean age was 69.2 ± 11.9 years for fracture cases and the mean age was 69.0 ± 11.7 years for control cases.

The BMD was measured by dual energy x-ray absorptiometry (Hologic^® ^4500 device) at lumbar spine and femoral neck. The mean lumbar spine BMD was respectively 0.814 ± 0.09 g.cm^-2 ^and 0.895 ± 0.16 g.cm^-2 ^(p < 0.05) for fracture cases and control cases. The mean femoral neck BMD was respectively 0.650 ± 0.09 g.cm^-2 ^and 0.686 ± 0.12 g.cm^-2 ^(ns) for the fracture cases and control cases.

### Study design

#### Image realization

This study was performed on trabecular bone radiographic images of the calcaneus. The calcaneus radiographic images were performed following a standardized procedure reported elsewhere [27]. Briefly, the X-ray tube voltage (48 kV), the exposure conditions (18 mAs) and the focal-calcaneus distance (1 meter) were fixed for all patients. Kodak single emulsion Min RG films were used and all developed by the same film processor at fixed developer and fixer temperatures.

**Figure 1:**
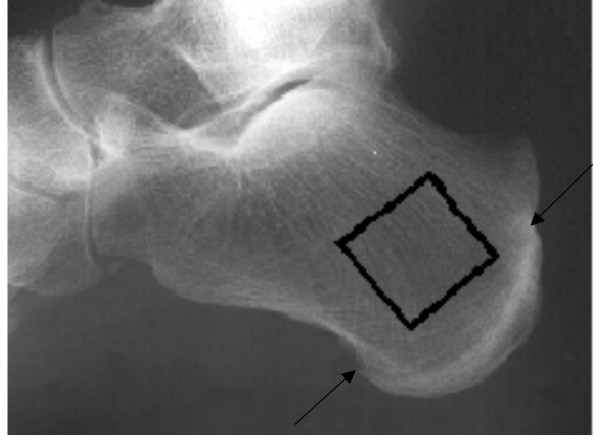
Radiographic image of the calcaneus showing the localization of the Region of Interest (ROI). Inside the ROI, longitudinal trabeculae correspond to the compressive trabecular network extended from the subtalar joint and the transverse to the tensile trabecular network sweeping backwards and upwards the great tuberosity. Arrows show anatomical landmarks corresponding to the bottom of the ROI.

In order to obtain digitized images of the calcaneus trabecular bone, the radiographic films were digitized with an AGFA Duoscan scanner (AGFA GEVAERT N.V., Morstel, Belgium) with 256 gray-levels. Calcaneus is known to be a heterogeneous site of trabecular bone [32]. For this reason we have selected a large region of interest (ROI) of 2.7 × 2.7 cm^2 ^(256 × 256 pixels with a pixel size of 105 μm). The ROI of 256 × 256 pixels (Figure 1) was defined from anatomic marks [27]. The basis of the ROI was positioned on a line linking the plantar aponeurosis insertion to the superior end of the Achilles tendon insertion, the ROI comprising both compressive and tensile trabecular network [10].

#### Noise filtering

The low frequency noise of an image corresponds to the gray-value variations over large distances, due to the radiological artifacts and to the fat tissues projections on the radiograph. In order to remove the low frequency noise and to take into account only trabecular components of the image, we used a convolution filter previously described by Geraets [33]. A kernel box was used; for each pixel, the average gray-value of the box was allocated to the box middle pixel. The new image obtained is the low-frequency image. The window must be small enough to extract the low frequency noise and large enough to prevent the trabecular pattern leaking into the low frequency region. For our images, the optimal size of the box was 25 × 25 pixels. The filtered image was obtained by subtracting the low-frequency image from the original images, this filter also removed cross of high intensity centered on the central pixel due to the finite bondaries of the ROI.

#### Fourier transform

The Fourier transform represents a signal in spatial frequency space. An image can be considered as a repartition of bright intensities in a (*xOy*) plane and can be expressed as a two-dimensional function *f*(*x*, *y*). The Fourier transform is expressed by a function *F*(μ,υ) with the two variables μ and υ corresponding to spatial frequencies in (μ *Oυ*) plane. The 2D Fourier transform spectrum of an image is expressed by the following formula:



where μ, υ and *x*, *y *are the respective variables of the frequency and the spatial domains and N the size of the image.

Any periodic structure in the original spatial-domain image is represented by peaks in the frequency-domain image at a distance corresponding to the period and a direction at right angle of the original orientation. If a mild degree of disorientation is introduced in an oriented periodic structure, the frequencies of the Fast Fourier transform (FFT) spectrum are spread over an angle corresponding to the deviation of the structure in the original image. By analogy, we hypothesized that the periodic structure is represented by trabeculae projection, and the degree of disorientation by anisotropy.

The magnitude of the transform corresponds to:



where *Re *= real part of the FFT and *Im *= the imaginary part of FFT.

The FFT was calculated on the gray level filtered images of the trabecular bone using the Visilog 5.1 software (Noesis). Then the magnitudes of the frequency images were divided to the total magnitude of the transform to normalize the contrast in the images according to the following formula:



#### Measurements on FFT spectrum

**Figure 2:**
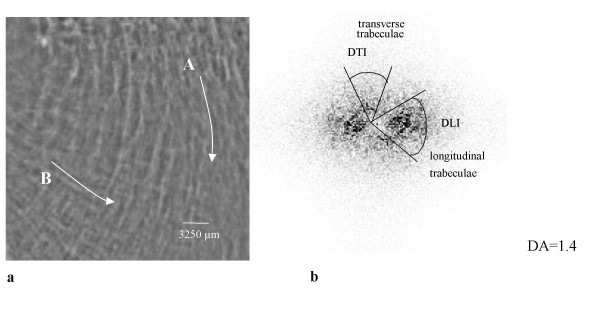
Control case **a**: ROI of the digitized radiographicimage of calcaneus trabecular bone. **b**: FFT spectrum **A**: corresponds to longitudinal trabeculae **B**: corresponds to transverse trabeculae DLI: Dispersion Longitudinal Index, spreading angle of the longitudinal trabeculae DTI: Dispersion Transverse Index, spreading angle of the transverse trabeculae

**Figure 3:**
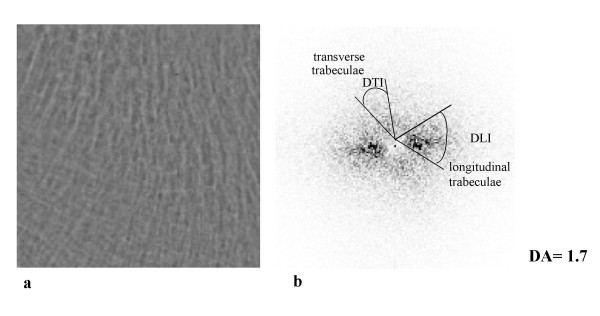
Vertebral fracture case **a **Region of Interest (ROI) of the digitized radiographic image of calcaneus trabecular bone. **b **FFT spectrum DLI : Dispersion Longitudinal Index, spreading angle of the longitudinal trabeculae DTI : Dispersion Transverse Index, spreading angle of the transverse trabeculae

Trabecular bone was assimilated to an oriented structure with two main directions, the ROI including longitudinal and transversal trabeculae (Figure 1). All the FFT spectra of the calcaneus radiographs involved horizontal and vertical conponents (Figures 2 and 3). The horizontal components were indicative of the longitudinal trabeculae fabric and the vertical components were indicative of the transverse trabeculae fabric. We have to notice that the so-called horizontal and vertebral trabeculae were so described on the ROI, which was slightly rotated by comparison to the original image. The limit between high energy area representing trabeculae and low energy area corresponding to the noise on the FFT spectrum were visually determined by the operator. We measured the spreading angles of the longitudinal and the transversal trabeculae named respectively the Dispersion Longitudinal Index (DLI) and the Dispersion Transverse Index (DTI). In order to obtain an average value, the DTI and DLI parameters were measured in the two symmetrical parts of the FFT spectrum conponents (Figures 2b and 3b).

An index relative to the trabecular fabric or degree of anisotropy was derived from the measured parameters DLI and DTI. The Degree of Anisotropy (DA) was defined as:



For a perfectly isotropic structure, the FFT spectrum has a disc shape and DA is equal to unity. More isotropic trabecular bone structures will have DA values closer to unity.

Figures 2 and 3 illustrate two examples of digitized radiographic images and FFT spectra of trabecular bone at calcaneus. Figure 2 corresponds to a control case and Figure 3 to a vertebral fracture case.

#### Synthetic images

**Figure 4:**
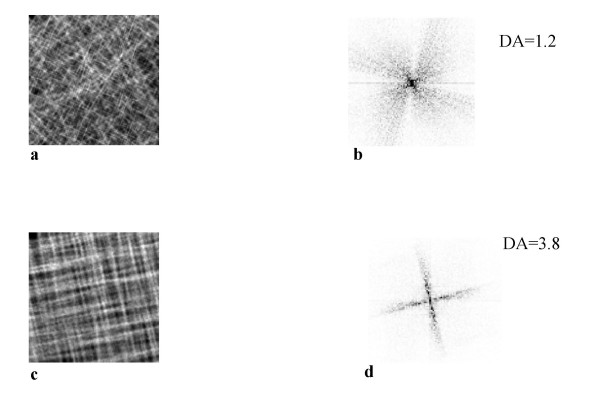
Synthetic images **a**: Synthetic image of astructure with orientations between 0 and 80° in 3 dimensional space **b**: Fast Fourier Transform (FFT) spectrum of the image 4a **c**: Synthetic image of a structure with orientations between 0 and 17° in 3 dimensional space **d**: FFT spectrum of the image 4c

To test the reliability of DA assessment, we tested this method on projected volume with known disorientations. We synthesized 6 series of 8 images composed of beam like structures more or less aligned following two directions in order to obtain structures close to trabecular bone of calcaneus. For the less anisotropic structure, horizontal and vertical disorientations varied from 0 to 80° (Figure 4a), and for the most anisotropic structure, the disorientation of the beam like structures varied from 0 to 17° (Figure 4c). These synthetic images comprised a wide variety of structures, from anisotropic to almost isotropic, a variety much wider than expected in the clinical X-rays. The projections of these volumes were analyzed with the same software as bone radiographs.

### Data analysis

#### Intra and inter-observer reproducibility of the measurements

The two measured parameters DLI and DTI and the derived parameter DA were determined to calculate the intra and inter-observer reproducibility. To determine the intra-observer reproducibility, a single observer performed two sets of measurements on the FFT spectra of 20 subjects with a one day interval between each set. To determine the inter-observer reproducibility, two sets of measurements were performed on the FFT spectra of 20 subjects by two observers. The observers were blinded for each sets of measurements.

The intra-observer and inter-observer reproducibilities were calculated for *n *subjects with the root mean square RMS average according to the following formula [34]:



where *SD*_*j *_is the standard deviation for the subject *j *and  is the average of the measurements for the subject *j*.

#### Clinical evaluation

Results in these two groups were compared using Student's *t*-test for comparisons of the means after checking Gaussian distribution. The area under ROC curves was calculated for BMD measurements and DA.

## Results

The results of synthetic images are represented on Table 1. The DA varied from 1.3 to.3.6, respectively corresponding from more anisotropic (Figures 4a and 4b) to almost isotropic structures (Figures 4c and 4d).

Table 2 shows the intra-observer and inter-observer RMSCVs. The RMSCVs of DA were respectively 1.5% and 3.1 %

Table 3 presents the results of the fabric indices obtained on the 22 osteoporotic fracture cases and 44 control cases. Results of DLI and DTI were statistically significant lower in osteoporotic cases (p < 0.01, p < 0.05, respectively) leading to a significantly higher DA.

In control cases DA was closer to 1 due to a large spreading of frequencies in the FFT spectra while DA was higher in osteoporotic cases in relation to a narrower frequency spreading. Comparing the DA values from Table 3 to Table 1, it appears that the controls (DA = 1.7) were in the 0–55° category and the fractures cases (DA = 1.9) were in the 0–45° category. The difference of the mean of DA between cases and controls was 8.7 % and close to the figured variation  corresponding to the 95% confidence interval of the measurements [35].

**Figure 5:**
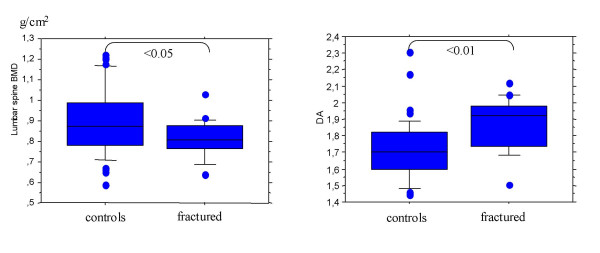
Box plots of lumbar spine BMD and DA. A T-test was used to compare controls and fractured patients.

Differences in DA determined by spectral analysis (p < 0.01) and lumbar spine BMD (p < 0.05) were significant (Figure 5). Whereas the difference between fracture cases and controls for femoral neck BMD was not significant. From the ROC analysis, the Area Under Curve (AUC) were respectively 0.65, 0.62, 0.64, 0.77 for lumbar spine BMD, femoral neck BMD, total femoral BMD and DA. There is a trend to higher AUC for DA comparatively to BMD measurements, but according to the small size of the population the statistical significance was not reached.

## Discussion

We have developed a new index of trabecular bone anisotropy on radiographs based on a spectral analysis of the gray-level images. In this pilot study, this new index applied to radiographs of the calcaneus has shown a reasonable reproducibility for a technique at its first step of development. As it was demonstrated with synthetic images, the DA of an almost isotropic structure is close to one and the DA of an anisotropic structure is greater than one. We hypothesized that the trabeculae did not randomly disappear, the effect might be a reduction of frequencies on the FFT spectra in preferential directions leading to an increase of anisotropy. The results of this pilot study involving 22 osteoporotic fracture cases and 44 controls suggest that the DA parameter may be potentially useful to distinguish fracture cases from control cases.

The main interest of this technique is that it can be applied directly to gray level radiographic images without previous binarization. The radiographic technique provides a projection image. However a good 2D-3D correlation has been described concerning microarchitecture [29] and anisotropy [18] from gray level analysis.

The significantly higher DLI and DTI in control cases suggest that there are more various orientations of the elemental structures around the two main orientations (longitudinal and transversal) in control cases than in osteoporosis cases with vertebral fractures. The larger range of orientations in controls corresponds to a lesser anisotropic structure than in osteoporosis. DTI was the best discriminant parameter between fracture patients and control cases but also the less reproducible. There are only few transversal trabeculae comparatively to longitudinal ones and they disappear first with osteoporosis due to a less contribution in bone strength. The difference between fracture cases and controls was close to errors attributed to the intra or inter-observer reproducibility. The intra-observer reproducibility is close to the 1 to 2% of reproducibility found in usual bone densitometry method as dual x-ray absorptiometry [37]. The poor inter-observer reproducibility is due to the difficulty to identify the limit between high energy area of the spectrum (corresponding to trabeculae) and low energy area (corresponding to noise) in relation to the few numbers of trabeculae projections. Since the population size is small, further studies are required with larger groups. We could expect more stringent results with a more homogenous population of fractured patients with vertebral fracture, for instance.

The higher anisotropy found in osteoporosis cases is in accordance with the findings of Newitt *et al*. [38] who report that the increase of bone resorption in osteoporosis leads to a loss of thinner trabeculae first, resulting in an increase of anisotropy. The study of Newitt *et al*. was performed with the MIL method on 3D magnetic resonance images at the radius. The MIL method is widely used to determine the trabecular 3D structure anisotropy but some authors [39,40] discuss its reliability since it reflects the boundary orientation rather than the real anisotropy of the structural elements.

This concept of transversal and longitudinal systems of trabeculae must be cautiously interpreted in our study. Indeed the ROI on the calcaneus radiographic images is tilted around 45 degrees (Figure 1). The trabeculae were named longitudinal and transversal in reference to the radiographic image (Figure 1) and not to the ROI orientation (Figures 2a and 3a). Longitudinal trabeculae correspond to the compressive trabecular network extended from the subtalar joint and the transverse to the tensile trabecular network sweeping backwards and upwards the great tuberosity [41].

The anisotropy of trabecular bone is different according to the skeletal sites: in a study comparing the properties of calcaneus, distal femur, proximal femur and vertebrae on human specimens, Majumdar *et al*. [17] found the highest anisotropy of trabecular bone at the calcaneus followed by distal femur and proximal femur, vertebrae constituting the least anisotropic site.

Our results corroborate the studies of Geraets *et al*. [33] on hip and radius, Wigderowitz *et al*. [24] on wrist radiographs, Mosekilde *et al*. [42] on vertebrae and Ciarelli *et al*. [43] on femoral head samples. The Line Fraction Deviation was developed by Geraets on binarized radiographic images, and leads to the determination of the preferential orientations. There was no calculation of an anisotropy index but it was possible to quantify the anisotropy from the deviation of polar diagram from a circle.

The lower values of the Line Fraction Deviation index found in osteoporotic subjects were consistent with the early loss of the secondary compressive trabecular group of the hip [44]. Moreover, Geraets *et al*. showed in a study of the distal radius that the Line Fraction Deviation values decreased along the transversal direction with age whereas the orientation along the axial direction remains stable during the entire life [33]. Wigderowitz *et al*. [24] found using the spectral analysis at the distal radius that the transversal trabeculae are preferentially absorbed, thinned and spared with age. Mosekilde *et al*. in vertebrae [42] showed that compressive strength was greater in the vertical direction than in the transversal direction. This anisotropy in vertebral strength increased with age and indicated according to the authors that the transverse trabeculae were selectively removed. Buck et al. have found that the oblique conponents declined in the cranio-caudal direction particularly for age superior to 60 years leading to variation in anisotropy [23]. Ciarelli *et al*. [43] also found that fracture cases have proportionally more trabeculae aligned along the primary load axis (and thus proportionally fewer transverse trabeculae) than control cases; the authors hypothesized that the loss of transverse trabeculae leads to the difference in anisotropy between groups. Zhao *et al*. characterized iliac trabecular bone by micro-QCT and showed that trabeculae thinning led to a more isotropic structure in the first postmenopausal years whereas the structure became more anisotropic in the later years [45]. They hypothesized that, in the later years, the remaining trabeculae would be more widely separated, less connected and some more thickened leading to an increase of anisotropy.

It has been well established that the calcaneus structure is heterogeneous [32,46]. Lin *et al*. [32] have studied the calcaneus microarchitecture on MRI images. They analyzed 20 to 25 ROIs 1 × 1 cm^2 ^in each calcaneus. They found a spatial heterogeneity in the posterior region of 40 %. In our study the ROI was 2.7 × 2.7 cm^2 ^and represented a much larger area; it was accurately defined by anatomic marks, this point avoiding large variation in fractal analysis [27]. Furthermore our ROI contained both transversal and longitudinal trabeculae, if transversal trabeculae disappeared, it should be possible to calculate DA and a high value will be obtained. As the DLI and DTI measurements were not yet automated, the parameters were measured in the two symmetrical parts of the FFT spectrum in order to obtain an average value. In a near future the automation of our method could be performed, allowing applications to large sets of images. In spite of this lack of automatization the reproducibility of the DA parameter was acceptable and allowed for the accurate characterization of osteoporosis changes in small series. At this first step of development, this clinical study must be considered as a preliminary study evaluating the potential of this anisotropy evaluation.

## Conclusion

This study has shown that the DA can be determined on plain radiographs using spectral analysis. The reproducibility of the DA values may be improved by automating the method. The distinction between fracture cases and control cases is very promising, but further studies are necessary to know if the DA evaluation could improve the osteoporotic fracture risk determination when combined with BMD and other textural parameters such as fractal analysis.

## Competing interests

The author(s) declare that they have no competing interests.

## Authors' contributions

BBI participated in the design of the study, carried out the measurements and the manuscript preparation, G L carried out measurements of reproducibility and synthetic images, CC performed statistical analysis and participated to the manuscript preparation, R H supervised the application of the Fourier Transform and CLB carried out the design of the study and supervised the manuscript preparation.

## Pre-publication history

The pre-publication history for this paper can be accessed here:



## Figures and Tables

**Table 1:** DA assessment on synthetic images composed of cylinders more or less aligned following two directions leading to isotropic and anisotropic structures.

Synthetic images						
Angles	0–80°	0–69°	0–52°	0–40°	0–28°	0–17°
DA	1.3	1.3	1.8	1.9	2.5	3.8

**Table 2:** Root Mean Square Standard Deviation Coefficient Variation RMSCV(%) for measured indices DLI and DTI and the degree of anisotropy.

	Intra-observer reproducibility	Inter-observer reproducibility
	
	CV(%)	CV(%)
Measured parameter		
DLI	2.4	3.4
DTI	2.4	4.1
Calculated parameter		
DA	1.5	3.1

DLI: Dispersion Longitudinal Index

DTI: Dispersion Transversal Index

DA: Degree of Anisotropy

**Table 3:** Fabric indices (mean ± SD) from the fabric parameters in osteoporotic fracture cases and control cases.

	Osteoporotic fractures	Controls	Statistical significance p
DLI	55.6 ± 6.2	59.1 ± 6.6	0.04
DTI	41.3 ± 5.9	46.8 ± 6.7	0.002
DA	1.87 ± 0.15	1.72 ± 0.18	0.001
